# Evaluation of a Commercial Mobile Health App for Depression and Anxiety (AbleTo Digital+): Retrospective Cohort Study

**DOI:** 10.2196/27570

**Published:** 2021-09-21

**Authors:** Margaret T Anton, Heidi Mochari Greenberger, Evie Andreopoulos, Reena L Pande

**Affiliations:** 1 AbleTo, Inc New York, NY United States

**Keywords:** digital mental health, mHealth, iCBT, coaching, depression, generalized anxiety, social anxiety, mobile phone

## Abstract

**Background:**

Digital solutions, such as web-based and mobile interventions, have the potential to streamline pathways to mental health services and improve access to mental health care. Although a growing number of randomized trials have established the efficacy of digital interventions for common mental health problems, less is known about the real-world impact of these tools. AbleTo Digital+, a commercially available mental health app for depression and anxiety, offers a unique opportunity to understand the clinical impact of such tools delivered in a real-world context.

**Objective:**

The primary aim of this study is to examine the magnitude of change in depression and anxiety symptoms among individuals who used AbleTo Digital+ programs. The secondary aim is to evaluate Digital+ module completion, including the use of 1:1 coaching.

**Methods:**

In this retrospective cohort study, we analyzed previously collected and permanently deidentified data from a consecutive cohort of 1896 adults who initiated using one of the three Digital+ eight-module programs (depression, generalized anxiety, or social anxiety) between January 1 and June 30, 2020. Depression, generalized anxiety, and social anxiety symptoms were assessed within each program using the Patient Health Questionnaire-9, the Generalized Anxiety Disorder-7, and the Social Phobia Inventory, respectively. Linear mixed effects models were built to assess the association between module completion and symptom change among users who completed at least four modules and had at least mild baseline symptom elevations, controlling for age, gender, and baseline symptom severity. Digital+ use, including module completion, 1:1 coaching calls, and in-app coach messaging, was also evaluated.

**Results:**

Significant effects were observed among depression (Cohen *d*=1.5), generalized anxiety (Cohen *d*=1.2), and social anxiety (Cohen *d*=1.0) program participants who completed at least four modules and had mild baseline elevations (n=470). Associations between module completion and change in depression (β=−1.2; *P*<.001), generalized anxiety (β=−1.1; *P*<.001), and social anxiety (β=−2.4; *P*<.001) symptom scores retained significance with covariate adjustment. Participants completed an average of 2.6 (SD 2.7) modules. The average total length of app use was 52.2 (SD 83.5) days. Approximately two-thirds of the users engaged in at least 1 coaching call (66.82%, 1267/1896) or in-app text messaging (66.09%, 1253/1896). Participants who completed at least four modules participated in significantly more coaching calls per module (mean 1.1, SD 0.7) than users who completed fewer than four modules (mean 1.0, SD 1.2; t_1407_=−2.1; *P*=.03).

**Conclusions:**

This study demonstrated that AbleTo Digital+ users experienced significant reductions in depression, generalized anxiety, and social anxiety symptoms throughout the program.

## Introduction

### Background

Effective mental health interventions exist for depression and anxiety [1,2], yet more than 50% of people in need of these services do not receive them [3]. A number of systemic- and individual-level barriers lead to these service gaps, including mental health workforce shortages, stigma, and financial barriers [4,5]. In addition, even when individuals are able to access traditional face-to-face services, the lag between symptom onset and treatment initiation is, on average, 11 years [6,7]. As the rates of anxiety and depression continue to increase [8], hastened in part by the current COVID-19 pandemic [9,10], access gaps are likely to worsen in an already strained, fragmented, and underresourced mental health care system [11]. These gaps in service have large societal, familial, and individual costs [12]. For example, in the United States, the estimated annual economic burden of depression alone is US $210 billion [13,14].

Digital solutions, including web-based and mobile behavioral health care interventions, have great potential to streamline pathways to mental health services and improve access to quality mental health care [15]. Stand-alone and adjunctive digital tools can provide timely mental health support that fits individuals’ daily lives from the comfort of their own homes. As such, these interventions have the potential to mitigate some of the most common barriers to mental health care, including transportation, time constraints, and stigma. In addition, digital interventions offer a cost-effective and scalable alternative to traditional treatment modalities.

A growing body of research supports the efficacy of digital interventions (ie, mobile interventions and web-based interventions), particularly for common mental health problems such as depression and anxiety [15-19]. Indeed, between 2009 and 2015, the National Institute of Mental Health awarded 404 grants to technology-enabled interventions, and more than 100 randomized trials have been conducted [20]. However, questions remain about the effectiveness of these tools, particularly in real-world settings [21], and their reach is often limited. Few mental health apps developed in the context of university-based research are being widely used, limiting their potential [22,23].

On the other hand, more than 10,000 commercially developed mobile apps focused on mental health are now widely available and easily accessible [24]. However, the vast majority of these tools lack rigorous testing or evaluation. A systematic review suggests that as few as 3% of the commercially available mobile mental health apps have any peer-reviewed evidence [25], and the apps most commonly downloaded and used contain minimal evidence-based content [23]. For example, Wasil et al [23] examined the proportion of evidence-based content based on monthly use data of common apps. They found that common treatment elements, such as exposure, reached only 2% of users. As a result, numerous efforts have been made to evaluate commercially available apps and create app repositories with standard reporting, and at least 45 frameworks have been developed to assist consumers in identifying the most effective and appropriate apps [24-26]. However, these efforts have fallen short. To date, personal searches on commercial app stores remain the most common method for finding mental health apps [27], and consumers are looking for new approaches to find these tools [28].

Partnering with health plans and employers to help disseminate these tools has the potential to help guide consumers to evidence-based apps that have the greatest potential for impact. There have been calls for health plans to incorporate digital solutions into their behavioral health resources and help their members navigate and discover apps that best meet their needs [29]. This approach may improve behavioral health care use by directing users to additional mental health services. In addition, employers are uniquely situated to link their employees to behavioral health resources. Adults in the United States spend most of their time at work, and mental health problems are often costly to employers because of lost workdays and decreased productivity. Preliminary research suggests a US $2-$4 return on investment for every dollar that employers spend on mental health resources [30]. However, little is known about the effects of these approaches.

### Objectives

There is a need to better understand the impact of digital interventions disseminated to real-world users in these new ways. AbleTo’s Digital+ program (AbleTo Digital+ has previously been reported on as Joyable [31]. Joyable, Inc, was acquired by AbleTo, Inc, in March 2019) provides a unique opportunity to understand the impact of such tools. Digital+ is a web- and mobile-based platform with personalized coaching for anxiety and depression made available to users through health plans and employer partners. Users are directed to one of three different programs based on their symptom presentation and goals. All Digital+ programs are based on cognitive behavioral principles and include psychoeducation, brief activities (<10 minute), weekly symptom tracking, and 1:1 coaching via phone and in-app messaging. Programs include (1) depression, an eight-module program focused on behavioral activation; (2) generalized anxiety, an eight-module program focused on worry exposures and distress tolerance; and (3) social anxiety, an eight-module program focused on exposure to feared social situations [31]. A critical next step is to better understand the impact of this tool on real-world users. The primary objective of this study is to examine the magnitude of changes in depression and anxiety symptoms among individuals who used the Digital+ programs. The secondary aim is to evaluate Digital+ use across the eight modules of each program and the use of 1:1 coaching.

## Methods

### Participants

In this retrospective cohort study, we analyzed previously collected and permanently deidentified data from a consecutive cohort of 1896 adults enrolled in an AbleTo Digital+ program between January 1 and June 30, 2020. Participants were aged ≥18 years and were required to have access to their own devices. Participants were excluded if they reported active suicidal ideation or psychosis. All study procedures were submitted to the Sterling institutional review board, Atlanta, Georgia, United States, and deemed exempt.

### Procedure

AbleTo, a technology-enabled virtual behavioral health care organization, partners with employers and health plans to make Digital+ accessible to employees and covered members in their networks. Employees and covered members are made aware of Digital+ through marketing campaigns and employer communications that explain the potential benefits of engaging in evidence-based, time-bound digital programs. A variety of engagement strategies were used to make individuals aware of the program and facilitate enrollment. These methods included divulging information about Digital+ on an employer’s benefits webpage, via posters, flyers, and table tents in office settings, and through email and text campaigns. Interested participants were then directed to access Digital+ via the web or a mobile app to complete a brief survey (approximately 2 minutes) to determine program appropriateness and create an account.

### Description and Structure of the Program

During enrollment, users completed a series of questions designed to assess their appropriateness for Digital+ and generate initial program recommendations. Screening questions to assess participant goals and primary presenting problems were used to determine which of Digital+’s three programs—(1) depression, (2) generalized anxiety, or (3) social anxiety—would best fit the users’ needs. Users had the opportunity to review the recommendation and select a different program if they felt that it was not the correct fit. Users then completed one of three standard assessments to establish baseline symptom severity and screening questions about suicidality and psychosis designed to assess safety and risk. Users were also asked to report any current or prior history of common, high-risk, comorbid psychiatric challenges (ie, substance use, eating disorders, bipolar disorder, and posttraumatic stress disorder) to further evaluate the clinical complexity.

Those deemed inappropriate for Digital+ (eg, active suicidal ideation and psychosis) were routed to various resources depending on their employer or health plan. These resources included, but were not limited to, the National Suicide Hotline, a Digital+ in-network provider matching service, or linkage to their health plan to assist in connecting individuals with a higher level of care. In addition, for some partners, individuals were offered the opportunity to enroll in a purely self-guided, five-module skills-based program if they did not wish to engage with a coach or in any of the primary Digital+ programs. Individuals who endorsed active suicidal ideation or psychosis and those in the five-module skills-based program were excluded from this analysis.

Digital+ programs included eight content modules, a 1:1 coaching option, and mood and symptom assessment. All psychoeducational materials and activities were based on core components of cognitive behavioral therapy (CBT), including cognitive restructuring, gradual exposure, and behavioral activation. Each module consisted of 4 to 6, approximately 10-minute activities, including deep breathing, progressive muscle relaxation, cognitive restructuring, and behavioral activation. The activities were organized to help users learn about CBT, depression, or anxiety; develop skills to challenge maladaptive thoughts; and practice newly acquired skills in real-life settings. Each module was designed to be completed within approximately 1 week, but users were not required to complete them in that amount of time. New activities become available once the previous activity is completed. Once the content was made available to users, they were able to revisit previous modules and activities and complete them as many times as desired. At the end of each module, users completed a standard assessment (refer to the *Measures* section for more information) before proceeding to a new module. Users were presented with feedback on their scores, and these scores were accessible to their coaches (see Table 1 for a description of the content of each program).

**Table 1:** Description of AbleTo Digital+ programs.

Module	Programs					
	Depression		Generalized anxiety		Social anxiety	
	Number of Activities	Content	Number of Activities	Content	Number of Activities	Content
1	6	Psychoeducation about depression Introduction to distress tolerance skills (eg, deep breathing)	6	Psychoeducation about stress and anxiety Introduction to distress tolerance skills (eg, deep breathing)	4	Psychoeducation about social anxiety Introduction to distress tolerance (eg, deep breathing)
2	4	Introduction to behavioral activation and planning for first activity	5	Introduction to automatic thoughts and cognitive distortions	4	Introduction to automatic thoughts and cognitive distortions
3	4	Introduction to automatic thoughts and cognitive distortions	4	Psychoeducation about avoidance and exposure Completion and review of first exposure activity	5	Psychoeducation about avoidance and exposure Completion and review of first exposure activity
4	3	Introduction to values-based behavioral activation, planning and completion of an activation exercise	5	Practicing cognitive skills, planning, and completing a worry-based exposure	4	Creating a fear hierarchy and planning and completing an exposure activity
5	4	Plan and complete an additional values-based activation	5	Plan and complete an additional exposure and mindfulness activity	4	Pick a new exposure from the fear hierarchy, plan, and complete the exposure.
6	4	Plan and complete an additional values-based activation	5	Plan and complete an additional worry-based exposure	4	Pick a new exposure from the fear hierarchy, plan, and complete the exposure
7	4	Plan and complete an additional values-based activation	5	Plan and complete an additional exposure and mindfulness activity	4	Pick a new exposure from the fear hierarchy, plan, and complete the exposure
8	6	Plan and complete an additional final activation Introduction to core beliefs and preparation for maintaining gains	5	Plan and complete final exposure Introduction to core beliefs and preparation for maintaining gains	4	Plan and complete final exposure Introduction to core beliefs and preparation for maintaining gains

### Coaching

All users were assigned a coach during program enrollment. Coaches are nonlicensed professionals with bachelor’s degrees and relevant work experience, course work, or certification in coaching. They receive intensive training in the principles of behavior change, motivational interviewing, Digital+ program content, and crisis intervention. Coaches are trained to focus on motivation and engagement by using motivational interviewing and validating the participants’ experiences with depression or anxiety. They also help users understand how program content and activities align with their individual goals, reinforce activity completion and skill use, and provide additional accountability through reminders and encouragement. Coaches are trained in crisis intervention and use the question, persuade, and refer method of crisis intervention. If a user needs a higher level of care, coaches will refer to external resources to better meet the user’s needs. Coaches receive ongoing supervision from a motivational interviewing certified trainer and one-on-one supervision to monitor competence in program knowledge and client service. A subset of coaching calls is reviewed to assess adherence to motivational interviewing protocols and monitor quality.

Users have the option to engage in coaching by scheduling an initial kickoff call with their coach during program enrollment, and a welcome message is sent to all users. Kickoff calls are 30 minutes and are used to orient users to the program, establish goals, and set expectations. If a user does not schedule a kickoff call during enrollment, coaches make three attempts to engage users. After three attempts with no contact, the proactive reach out from the coaches is suspended. Users, however, continue to have access to coaches and can schedule calls or message coaches throughout the program, regardless of their initial preference for coaching, and coaches respond within one business day. Subsequent weekly 15-minute coaching calls are available to users throughout the eight-module program and focus on reviewing activities, clarifying goals, and encouraging ongoing use. Users also have access to in-app text messaging.

### Measures

#### Participant Characteristics

Consistent with the standard program experience, users provided basic demographic information (ie, age and gender) and indicated any current or recent serious behavioral health concerns by selecting all that applied from a list of common comorbid mental health problems, including substance use, bipolar disorder, eating disorder, and posttraumatic stress disorder.

#### Outcome Measures

Participants in the depression program completed the Patient Health Questionnaire-9 (PHQ-9) [32] during enrollment and after completion of each module. The PHQ-9 is a 9-item self-report measure designed to evaluate the presence of depressive symptoms during the past two weeks. Items are rated on a scale ranging from 0 (*not at all*) to 3 (*nearly every day*). Total scores range from 0 to 27, and cut-off scores for mild, moderate, moderately severe, and severe depressive symptoms are 5, 10, 15, and 20, respectively.

Participants in the generalized anxiety program completed the Generalized Anxiety Disorder-7 (GAD-7) scale [33] during enrollment and after completion of each module. The GAD-7 is a 7-item self-report measure of anxiety. Items are rated on a scale ranging from 0 (*not at all*) to 3 (*nearly every day*). Total scores range from 0 to 21, and the cut-off scores for mild, moderate, and severe anxiety symptoms are 5, 10, and 15, respectively.

Participants in the social anxiety program completed the Social Phobia Inventory (SPIN) [34] during the enrollment and after completion of each module. The SPIN is a 17-item self-report questionnaire that assesses social anxiety symptoms during the past week. Items are rated on a scale ranging from 0 (*not at all*) to 4 (*extremely*). Total scores range from 0 to 68, and the cut-off scores for mild, moderate, severe, and very severe are 20, 31, 41, and 51, respectively [35,36].

#### Digital+ Use

Patterns of Digital+ activity and module completion were explored using passive data collected through a digital platform. These data included whether the modules were started and/or completed. Time spent using Digital+ was assessed by examining the dates of the first and last activity use. In addition, patterns of coaching use were evaluated using the number of coaching calls completed per module and the number of incoming messages per module.

### Statistical Analysis

Baseline demographic and clinical characteristics were reported as frequencies and percentages for categorical variables and means and SDs for continuous variables. Outcome measures throughout time were reported as means and SDs. Baseline characteristics were compared between those who completed all eight modules and those who discontinued use before completion using a two-sample, two-tailed *t* test for continuous variables and chi-square tests for categorical variables. Linear mixed models with a random intercept and slope were constructed using restricted maximum likelihood estimation procedures to examine the association between module completion and depression, anxiety, and social anxiety symptom change throughout the Digital+ programs.

First, models were estimated separately for each outcome measure, and the covariance structure that provided the best model fit was identified. On the basis of model fit indices, the best-fitting model was carried forward into the covariate models to examine the associations between baseline participant characteristics (ie, baseline symptom severity, age, and gender) and changes in symptom severity throughout time. Participants with at least mild symptom severity at baseline (ie, PHQ-9 ≥5, GAD-7 ≥5, or SPIN ≥20) who completed at least four modules were included in these analyses. Consistent with established standards for internet-based interventions [37], users who had completed four modules were included because they were exposed to all primary program content at this point (ie, psychoeducation, cognitive restructuring, and practice activities). In addition, we examined within-subject effect sizes and the association between module completion and symptom reduction using linear mixed models among users who enrolled with at least mild baseline symptom elevations, regardless of intervention exposure (n=1694). Digital+ module completion, completed coaching calls, and incoming and outgoing messages were reported using frequencies and means. The association between module completion and baseline scores was assessed using unadjusted linear regression. Program retention or time spent using Digital+ was defined as the number of days between completing the first and last recorded activities. All analyses were conducted using SAS 9.4.

## Results

### Participants

A total of 1896 participants were enrolled and initiated the use of Digital+ between January 1, 2020, and June 30, 2020. The baseline characteristics of the users are shown in Table 2.

**Table 2:** Baseline sample characteristics (N=1896)^a^.

Variables			Total (N=1896)		Depression (n=560)		Generalized anxiety (n=974)		Social anxiety (n=362)
Age (years), mean (SD)			37.4 (11.8)		37.1 (12.2)		38.2 (11.7)		35.9 (11.6)
**Gender, n (%)**									
	Female	1196 (63.1)		356 (63.6)		636 (65.4)		204 (56.4)	
	Male	470 (24.8)		147 (26.3)		231 (23.7)		92 (25.4)	
	Nonbinary	14 (0.7)		7 (1.3)		2 (0.2)		5 (1.4)	
	Not disclosed	215 (11.3)		50 (8.9)		104 (10.7)		61 (16.9)	
	Missing	1 (0.1)		0 (0)		1 (0.1)		0 (0)	
**Source, n (%)**									
	Employer	1359 (71.7)		399 (71.3)		667 (68.5)		293 (80.9)	
	Health plan	537 (28.3)		161 (28.8)		307 (31.5)		69 (19.1)	
**Patient-reported psychiatric history^a^, n (%)**									
	Substance use	29 (1.5)		15 (2.7)		4 (0.4)		10 (2.8)	
	Eating disorder	145 (7.6)		61 (10.9)		56 (5.8)		28 (7.7)	
	Bipolar disorder	83 (4.4)		36 (6.4)		31 (3.2)		16 (4.4)	
	Passive suicidal ideation	95 (5)		44 (7.9)		31 (3.2)		20 (5.5)	
	Posttraumatic stress disorder	117 (6.2)		39 (7)		57 (5.9)		21 (5.8)	

^a^Individuals could endorse more than one psychiatric difficulty. In total, 17.51% (332/1896) of users reported at least one current or recent psychiatric challenge.

Of those enrolled, 29.54% (560/1896) enrolled in the depression program, 51.37% (974/1896) enrolled in the generalized anxiety program, and 19.09% (362/1896) enrolled in the social anxiety program. At baseline, 95.2% (533/560) of users met the criteria for at least mild depression on the PHQ-9 (ie, total score ≥5). The criteria for at least mild anxiety on the GAD-7 scale (ie, total score ≥5) were met by 90.3% (879/974) of users in the generalized anxiety program. The criterion for social anxiety (ie, total score ≥20) was met by 77.9% (282/362) of users in the social anxiety program.

Users were asked to select any current or recent psychiatric concerns from a list of common comorbidities during program enrollment. At least one current or recent psychiatric disorder was endorsed by 17.51% (332/1896) of users. Users who endorsed at least one recent or current psychiatric concern had significantly higher baseline PHQ-9 (14.8 vs 11.8; *P*<.001), GAD-7 (14.2 vs 11.0; *P*<.001), and SPIN (35.7 vs 30.0; *P*=.002) scores than users who did not endorse any recent or current psychiatric concerns.

### Clinical Outcomes

Descriptive statistics for the outcome measures throughout time are summarized in Table 3 for all the users.

Among users who completed at least half of the program content and had mild baseline symptom elevations (n=470), there were significant reductions in PHQ-9, GAD-7, and SPIN scores. These reductions corresponded to large within-group effect sizes across all three programs among those who completed at least four modules. The effect sizes for depression, generalized anxiety, and social anxiety symptoms were 1.5, 1.2, and 1.5, respectively. Similarly, the within-group effect sizes among those participants who completed all eight modules were large across all three programs. The effect sizes for depression, generalized anxiety, and social anxiety symptoms were 1.7, 1.7, and 1.4, respectively.

Of those who completed at least four modules in the depression program and had at least moderate depressive symptoms at baseline (PHQ-9 ≥10; mean 15.0, SD 3.6), 75.7% (87/115) of participants met the criteria for treatment response (PHQ-9 <10; mean 6.6, SD 4.8). In addition, 73.9% (122/165) of participants in the generalized anxiety program who completed four modules and had at least moderate symptoms (GAD-7 ≥10) at baseline (mean 15.0, SD 3.1) met the criteria for response (GAD-7 <10) at their last assessment (mean 7.0, SD 4.6). For the social anxiety program, among users who completed at least four modules and had SPIN scores of 20 and higher at baseline (mean 37.0, SD 11.0), 42% (31/74) of participants achieved a response (SPIN<20) by their last assessment (mean 24.9, SD 14.5).

**Table 3:** Symptom severity by module completed (N=1896).

Outcome	Module 1 (N=1896)	Module 2 (n=976)	Module 3 (n=700)	Module 4 (n=525)	Module 5 (n=430)	Module 6 (n=343)	Module 7 (n=305)	Module 8 (n=267)	Users who completed ≥4 modules			Users who completed all 8 modules	
									Effect size (Cohen *d*)	*P* value	Effect size (Cohen *d*)		*P* value
Depression, n (%)	560 (29.5)	273 (48.8)	230 (41.1)	181 (32.4)	157 (28)	135 (24.1)	119 (21.3)	102 (18.2)	N/A^a^	N/A	N/A		N/A
PHQ-9^b^, mean (SD)	12.5 (5.6)	9.9 (5.3)	8.4 (4.9)	7.20 (4.8)	6.3 (4.9)	5.5 (4.7)	5.0 (5.0)	4.8 (4.8)	1.5	<.001	1.7		<.001
PHQ-9 >4, n (%)	533 (95.2)	231 (84.6)	177 (77)	122 (67.4)	94 (58.9)	64 (47.4)	49 (41.2)	33 (32.3)	N/A	N/A	N/A		N/A
PHQ-9 >9, n (%)	368 (65.7)	130 (47.6)	77 (33.5)	46 (25.4)	29 (18.5)	22 (16.3)	18 (15.1)	13 (12.8)	N/A	N/A	N/A		N/A
Generalized anxiety, n (%)	974 (51.4)	500 (51.3)	342 (35.1)	248 (25.5)	206 (21.2)	159 (16.3)	136 (14)	126 (12.9)	N/A	N/A	N/A		N/A
GAD-7^c^, mean (SD)	11.4 (5.2)	10.13 (4.9)	8.14 (4.2)	7.4 (4.3)	6.87 (4.3)	6.59 (4.5)	5.1 (3.8)	4.7 (3.6)	1.2	<.001	1.7		<.001
GAD-7 >4, n (%)	879 (90.3)	432 (86.4)	270 (79)	180 (72.6)	141 (68.5)	101 (63.5)	76 (52.8)	61 (48.4)	N/A	N/A	N/A		N/A
GAD-7 >9, n (%)	589 (60.5)	262 (52.4)	116 (33.9)	67 (27)	44 (21.4)	32 (20.1)	16 (11.1)	13 (10.3)	N/A	N/A	N/A		N/A
Social anxiety, n (%)	362 (19.1)	203 (56.1)	128 (35.3)	96 (26.5)	67 (18.5)	51 (14.1)	40 (11.1)	39 (10.8)	N/A	N/A	N/A		N/A
SPIN^d^, mean (SD)	31.1 (13.8)	31.2 (13.9)	26.1 (14.9)	24.8 (14.3)	21.5 (13.4)	18.9 (11.8)	16.7 (11.8)	14.7 (11.6)	1.0	<.001	1.7		<.001
SPIN >19, n (%)	282 (77.9)	155 (76.4)	77 (60.2)	58 (60.4)	34 (50.8)	22 (43.1)	15 (37.5)	11 (28.2)	N/A	N/A	N/A		N/A

^a^N/A: not applicable.

^b^PHQ-9: Patient Health Questionnaire-9.

^c^GAD-7: Generalized Anxiety Disorder-7.

^d^SPIN: Social Phobia Inventory.

**Figure 1:**
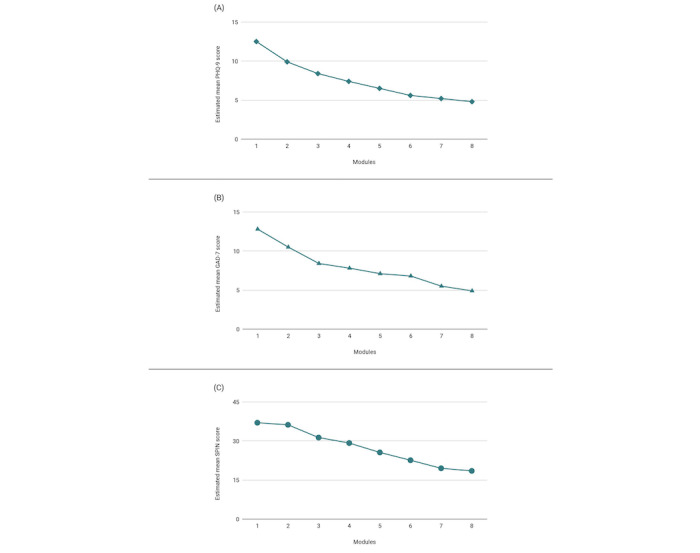
Estimated mean outcome scores by module for users who completed at least four modules. (A) depression program; (B) generalized anxiety program; and (C) social anxiety program. GAD-7: Generalized Anxiety Disorder-7; PHQ-9: Patient Health Questionnaire-9; SPIN: Social Phobia Inventory.

Model-implied estimates for PHQ-9, GAD-7, and SPIN scores by program module are presented in Figure 1. In the linear mixed model to evaluate associations between depression program module completion and symptom reduction among users with at least mild baseline symptom severity (PHQ-9 ≥5) and exposure to at least four modules (n=169), significant fixed effects of module completion (*F*_1972_=319.4; *P*<.001) and baseline PHQ-9 scores (*F*_1164_=454.9; *P*<.001) but not age (*F*_1164_=1.2; *P*=.19) or gender (*F*_2164_=0.82; *P*=.44) were observed. There was also significant variability around the mean intercept (*P=*.001) and slope (*P*<.001) and significant residual variance (*P<*.001).

Similarly, there were significant fixed effects of anxiety program module completion (*F*_11,258_=332.5; *P*<.001) and baseline GAD-7 scores (*F*_1222_=492.2; *P*<.001) but not age (*F*_1222_=0.17; *P*=.68) or gender (*F*_2222_=0.40; *P*=.67) on changes in symptom severity throughout time among users who had at least mild baseline generalized anxiety disorder symptoms (GAD-7 >5) and completed at least four modules of content (n=227). There was also significant variability around the mean intercept (*P*=.02) and slope (*P*<.001) and significant residual variance (*P*<.001).

In the linear mixed effects model among social anxiety program users with elevated baseline symptoms (SPIN >20) exposed to four or more digital modules (n=74), there were significant fixed effects of module completion (*F*_1366_=114.9; *P*<.001), baseline SPIN scores (*F*_169_=326.3; *P*<.001), and age (*F*_169_=9.2; *P*=.004), but not gender (*F*_269_=1.3; *P*=.27). There was also significant variability around the mean intercept (*P*=.02) and slope (*P*<.001), and there remained significant residual variance (*P*<.001).

To understand the impact of the intervention as delivered, we examined primary outcomes among all users who enrolled with at least mild baseline symptom elevations, regardless of intervention exposure (n=1694). The within-person effect sizes were 0.58, 0.50, and 0.44 for depression, generalized anxiety, and social anxiety symptom scores, respectively. There was a significant association between the number of modules completed and symptom reduction on the PHQ-9 (*P*<.001), GAD-7 (*P*<.001), and SPIN (*P*<.001) scores. Across all three programs, higher baseline symptom severity was significantly associated with slower symptom reduction (*P*<.001). There were no significant relationships among age, gender, and symptom reduction.

### Digital+ Use

Of those who enrolled, 27.9% (529/1896) completed at least half of the program content (ie, four modules), and 13.08% (248/1896) completed all eight modules in their respective programs. Among users who completed all eight modules in one program and those who did not, there were no significant differences in mean age (38.5 vs 37.3 years; *P*=.13), gender (26.21% female (313/1196) vs 24.59% (116/470) male; *P*=.14), or mean baseline scores on the GAD-7 (12.1 vs 11.3; *P*=.14) or SPIN (30.1 vs 31.2; *P*=.66). However, there were significant differences in baseline PHQ-9 scores (*P*=.01) between individuals who completed all program content (mean 11.2, SD 5.1) and those who discontinued (mean 12.8, SD 5.7).

Digital+ users completed 2.6 (SD 2.7) modules on average. Participants in the depression program (mean 2.9, SD 3.0) completed significantly (*F*_2_=4.2; *P*=.01) more modules than those in the generalized (mean 2.5, SD 2.7) and social anxiety (mean 2.5, SD 2.4) programs. On average, the length of time from first to last activity was 52.2 (SD 83.5) days, and of the 1896 participants who initiated the use of Digital+, 45.09% (855/1896) were retained at 30 days and 26.85% (509/1896) were retained at 60 days.

Higher baseline depressive symptomatology was associated with fewer modules completed (β=−.1; *P*=.02). However, higher baseline generalized anxiety symptomatology was associated with more modules completed (β=.1; *P*=.01). Gender was also significantly associated with the number of modules completed (*F*_3_=4.3; *P*=.01), with women completing 2.7 modules (SD 2.7) and men completing 2.6 modules (SD 2.7) on average. However, there was no significant relationship between the number of modules completed and baseline SPIN scores, age, or endorsing at least one recent or current psychiatric concern.

### Coaching

Overall, 66.82% (1267/1896) of participants engaged in at least one coaching call. On average, users scheduled 3.8 (SD 4.4) calls and completed 3.1 (SD 4.2) calls throughout their programs. Those who completed at least four modules (mean 1.1, SD 0.7) completed significantly more calls per module than those who completed fewer than four modules (mean 1.0, SD 1.2; t_1407_=−2.1; *P*=.03). In addition, users who completed at least one coaching call (mean 3.4, SD 2.8) completed significantly more modules than users who never engaged in coaching calls (mean 1.0, SD 1.5; t_1894_=−20.3; *P*<.001).

There was a significant relationship between age and the number of calls completed, such that older participants completed more calls than younger participants (β=.02; *P*=.01). In addition, baseline depression (β=−.1; *P*=.03) and generalized anxiety (β=.1; *P*=.01) symptom severity scores were associated with the number of calls completed. In the depression program, users with higher baseline levels of depressive symptomatology completed fewer calls than those with lower baseline symptomatology. In contrast, in the generalized anxiety program, users with higher baseline levels of anxiety symptoms completed more calls than those with lower baseline symptom severity. There were no significant associations among program (*P*=.21), gender (*P*=.16), or current or recent psychiatric difficulties (*P*=.89); baseline social anxiety symptom severity (*P*=.92); and the number of calls completed.

In-app messaging was used by most participants, with 66.09% (1253/1896) of users sending at least one in-app coach text message and an average of 4.5 (SD 11.1) messages sent to coaches. Users who completed fewer than four modules sent approximately one message to every seven messages coaches sent, whereas users who completed at least four modules sent approximately one message to every four messages from coaches (14.28% vs 25.75%; *P*<.001). There were no significant associations between program (*P*=.13), gender (*P*=.51), baseline depression (*P*=.68), or social anxiety (*P*=.76) symptom severity and the number of messages sent to coaches. However, there was a significant relationship between baseline generalized anxiety symptom severity and the number of messages users sent to coaches, such that higher baseline anxiety was associated with more sent messages (β=.2; *P*<.001). Age was also significantly related to the number of messages sent, such that older users sent coaches significantly fewer messages than younger users (β=−0.1; *P*=.03). Finally, there was also a significant relationship between user-endorsed recent or current psychiatric concerns and the number of incoming messages from users (*F*_1_=4.24; *P*=.04), such that those who endorsed at least one concurrent psychiatric challenge sent coaches significantly more messages (mean 5.6, SD 10.4) than those who did not report any recent or current psychiatric concerns (mean 4.2, SD 11.3).

## Discussion

### Principal Findings

This study adds to a small yet growing body of literature examining the magnitude of symptom reduction among users of commercially available digital mental health apps in a real-world context. Overall, Digital+ programs demonstrated significant reductions in depressive, generalized anxiety, and social anxiety symptoms throughout the program. The magnitude of these effects appeared to grow as more modules were completed; however, users who completed at least half of the program content also experienced significant and large reductions in symptomatology. These improvements were largely consistent across participant characteristics (ie, age and gender). In all three programs, those with more severe baseline symptomatology experienced smaller symptom reductions than those with lower baseline symptom severity. Of the 1896 AbleTo Digital+ users who initiated use between January 1, 2020, and June 30, 2020, 27.90% (529/1896) completed half of the program content, and 13.08% (248/1896) completed all the program content, with a 30-day retention rate of 45.09% (855/1896). Completion rates were consistent across age, gender, and baseline generalized and social anxiety symptom severity. Users with more severe baseline depressive symptomatology completed fewer modules than those with lower baseline scores. Almost all Digital+ users endorsed interest in 1:1 coaching, and approximately two-thirds of users engaged in at least one coaching call (66.2%, 1267/1896) and/or in-app text messaging (66.09%, 1253/1896). Users who completed at least four modules completed significantly more coaching calls per module than users who completed fewer than four modules.

### Comparison With Previous Work

Regarding clinical outcomes, the magnitude of symptom reduction among participants in Digital+ programs was comparable with or larger than those seen in the broader literature for internet-based CBT [38-40] and similar digital products for anxiety and depression [41,42]. For example, a recent meta-analysis suggests that in randomized trials, smartphone apps had moderate positive effects on depression [18] and anxiety symptomatology [17]. Also consistent with broader literature, these findings suggest comparable outcomes across several key demographic variables, including age and gender. Thus, this study adds to the growing body of literature supporting the efficacy of these interventions for a broad range of users [18]. Notably, the magnitude of symptom improvement is similar to effect sizes seen in studies of face-to-face CBT [43,44], although it is important to note that individuals engaging in Digital+ may differ from those presenting to face-to-face treatment.

AbleTo Digital+’s completion rate was comparable with or higher than that reported in a previous meta-analysis of similar digital interventions (ie, 0.5%-28.6%) [45], and the 45% 30-day retention rate was approximately 15 times higher than the 3.3% 30-day retention rate previously reported for other commercial mental health apps without human support, which is known to be key for improving engagement [46]. Although the use and retention rates were relatively high compared with other commercially available apps, there was still a substantial drop off, particularly earlier in the programs. For digital interventions to reach their full potential, optimizing engagement is necessary and remains a top research priority [47,48]. We hypothesized several reasons for premature dropouts. First, Digital+ may not be the appropriate intervention for some individuals, and they prematurely drop out because the intervention did not meet their needs. A key priority should be to link individuals to the appropriate level of care, and strategies are needed to link users to appropriate services. Digital+ coaches were trained to assist users in finding a more appropriate level of care if needed by linking them to their health plan resources or a Digital+ in-network provider matching service, which helped users locate and schedule appropriate care. Alternatively, it is possible that some individuals left the program owing to feeling better or what has been termed *happy abandonment* [49]. Although some Digital+ users experienced significant symptom reduction when completing only half of the program content, the long-term impact of premature dropout among these individuals remains unknown. Finally, given the finding that individuals with more severe baseline depression were more likely to discontinue using Digital+, motivation may be a primary target for sustained engagement. Future research is required to explore the reasons for and predictors of premature dropout, including both user- and program-related factors and the clinical implications of premature dropout.

Human support has consistently been shown to improve engagement in and sustained use of digital interventions [50-52]; however, much remains unknown about the aspects of coaching most closely linked to improved engagement [53]. Research has demonstrated that coaching focuses on reminding users to engage, and providing personalized feedback on completed content can boost engagement [53-55]. However, questions remain about the timing, intensity, and structure of coaching associated with higher engagement. More intensive coaching earlier in Digital+ programs may be needed to decrease premature dropout, particularly among groups of users who are more prone to drop out. In addition, one-third of the users did not engage in 1:1 coaching. Given the relationship between coaching and engagement, strategies are needed to promote coaching initiation among those most likely to benefit from additional support. Future research is also necessary to better understand the impact of coaching interactions on engagement and outcomes and develop and test tailored coaching strategies, including testing different coaching techniques, methods, and doses.

Although users with a broad range of symptom presentations benefited from Digital+, there was a consistent finding across programs that those with more severe baseline symptomatology experienced less improvement throughout the programs. In the case of depression, these individuals were also more likely to discontinue use prematurely. These findings are consistent with and add to the randomized controlled trial literature suggesting that digital interventions have the most consistent benefit for those experiencing mild to moderate symptoms at the time of initiation [18,56]. As digital mental health interventions continue to evolve, it is critical that we better understand who is most likely to benefit from these interventions to direct patients to the appropriate level of care and optimize outcomes.

Several stepped and staged care models that incorporate digital interventions into the broader behavioral health care system are emerging. For example, the United Kingdom’s National Health System’s Improving Access to Psychological Treatments [57] and Australia’s Mindspot Clinics [58] have integrated computerized and digital interventions into their suite of treatment options to improve efficiency and access to care. In both of these systems, digital interventions are considered first-line interventions that can be effective for those with mild to moderate symptoms and serve as a gateway to additional or more intensive interventions, if needed. Incorporating interventions such as Digital+ into the behavioral health care system offerings has the potential to reserve more intensive interventions for those who need them the most, decrease wait times, and mitigate worsening workforce shortages. As we learn more about who is likely to engage and benefit, we can use data-driven approaches combined with personal preferences to direct people to timely and appropriate services.

Finally, despite a growing body of literature supporting the efficacy of these interventions and consumer interest, consumers continue to struggle to identify high-quality, evidence-based products [27,28]. Novel approaches to disseminating tools such as Digital+ are needed to help people identify the tools best supported by research evidence that are most likely to help them. Research suggests that the interest in digital mental health interventions outpaces uptake and that individuals desire easier access to information on effective tools and look to trusted providers for information on which tools to use [28]. Health plans and employers may be uniquely situated to help streamline the dissemination of these tools and direct users to the best possible services by adding them to their suite of behavioral health care offerings. This approach can boost awareness and enhance credibility. This study demonstrates the potential of this approach; however, future research is needed to continue to evaluate the impact of these dissemination methods, particularly concerning improving access and decreasing health care and workplace costs.

### Limitations

The results of this study must be interpreted in light of some limitations. First, the lack of a control group means that we were unable to account for the natural remission of symptoms, the effect of missing data, or fully understand the impact of 1:1 coaching on engagement and outcomes. This limitation, however, was mitigated to some extent by the weekly collection of symptom severity scores and conservative statistical modeling that incorporated those who did not complete all intervention content [59]. In addition, the real-world context of this study is seen as a primary strength in that it establishes proof of concept for a health plan and employer rollout of mental health apps while establishing clinical impact.

Second, only a small number of baseline characteristics were collected to minimize user burden. However, this limited our ability to evaluate a broader set of factors that might be related to engagement and treatment response. The results suggest that above and beyond the known participant characteristics (eg, age, gender, and baseline symptom severity), there was significant variability unaccounted for in both engagement and treatment response. Although age and gender were not associated with treatment response, indicating the potential broad applicability of Digital+, additional information on participant demographics, including race or ethnicity and socioeconomic status, may help us better understand who is most likely to benefit. In addition, other data such as treatment history, concurrent treatment, and psychiatric medication use were not available. Collecting these data in the future would allow us to better understand how psychiatric treatment history affects program retention and response and potentially inform the integration of Digital+ into staged or stepped care models.

Third, these analyses precluded examining the impact of suicidal ideation and/or psychosis on outcomes, app use, or 1:1 coaching. Future research is needed to better understand the impact of these risk factors. Fourth, all users needed access to a device (eg, smartphone, tablet, or computer) and internet access to participate in Digital+. Although 81% of adults in the United States own a smartphone [60], this may limit the generalizability of these results to individuals who do not have access to a device or the internet. Finally, participants were not followed beyond the program period, making it difficult to draw conclusions about the sustained impact of Digital+ programs. This may be particularly important for users who discontinued use before full program completion yet showed symptom improvement. Future research is needed to understand the long-term clinical impact of tools such as Digital+.

### Conclusions

This study demonstrated that Digital+ users experienced significant reductions in depression, generalized anxiety, and social anxiety symptoms throughout the programs, independent of user age, gender, and baseline symptom severity. Overall, 30-day retention rates were significantly higher than previously reported rates for other commercially available mental health apps, particularly self-guided ones. In addition, users who completed at least half of the program content completed more 1:1 coaching calls than users who completed fewer than half of the program content. Participants who enrolled in Digital+ through their employees and health plan benefits experienced clinically significant symptom reductions. Digital+ may offer a scalable, low-cost additional service that may help mitigate workforce shortages and other common barriers to treatment. Future research is needed to continue to identify those who are most likely to benefit from these apps and examine how best to integrate digital interventions such as Digital+ into the broader behavioral health care system.

## Abbreviations

CBT: cognitive behavioral therapy
GAD-7: Generalized Anxiety Disorder-7
PHQ-9: Patient Health Questionnaire-9
SPIN: Social Phobia Inventory
